# Kind Words

**Published:** 1876-03

**Authors:** 


### Editorial and Miscellaneous.

ALWAYS read our advertisements.

ALL communications, of every character, must be addressed to Dr. Thomas S. Powell, 86 Pryor street.

SEND money by check, postal order, or registered letter. We are willing to endorse for the honesty of our subscribers, but cannot be responsible for irregularity of the mails, or forgetfulness of friends. Address all to Dr. T. S. Powell.

WRITE your name, post-office, county and State plainly. Be sure to say to which post-office you wish THE RECORD sent, and always date your letters and head them with your own post-office.

WE receive many well written letters, but doubt that the authors are able to decipher their signatures. Communications for publication must be plainly and carefully written, on only one side of the paper. Don't forget this.

WE publish in this issue the address of Rev. Mr. Evans, and Dr. Adams, made at the commencement exercises of the Georgia Medical College at Augusta. It is not our custom to devote the space of THE RECORD to public addresses, but in the present instance we pnblish them because we think they are calculated to accomplish good to the profession. In our next issue, if space permits, we propose to submit some thoughts suggested to our mind by reading these addresses.

WE invite attention to the article of Dr. Baxter, in the present number. The Doctor is a progressive and accomplished gentleman, and invites discussion. We hope that as THE RECORD is the medium through which the profession is invited to interchange opinions, that any one entertaining different views, will present them for publication.

OUR associate editors, co-laborers, and the profession generally, are requested to send up matter for our "Original Department." Let your articles be brief, pointed and practical. We especially desire reports of cases which illustrate new and useful plans of treatment. Also discoveries in the field of therapeutics, and valuable recipes. Send up your contributions, friends, and let us push forward the cause of medical progress, and do something to promote the good of the profession to which we belong.

*Kind Words.*—If we had space we could publish a number of private letters of an encouraging nature to our enterprise, in the hope that it would exert upon our readers, (as it has upon us,) a stimulating influence in fostering medical literature, and working for THE RECORD. At present we can only give place to the following:

PITTSBORO', February 23, 1876.

*My Dear Doctor:* * * * I know of no man whose hand I would sooner grasp than yours. This may never happen in this life, but I trust we may meet in Heaven. * * *

There will be an artist of some celebrity here soon, and I will procure a photograph and send it to you, and in return shall expect yours, for if I never see your physiognomy in *propria personæ*. I want to see it photographed. Doctor, I am getting old; have been in the harness forty years. * * * Still I love my profession, and expect to die in the traces. * * * I shall soon be sixty-four years old, and I fear failing fast. How old are you? * * * You will please find enclosed six dollars, for which you will send this volume of THE RECORD to Dr. R. R. Ihric, Pittsboro', N. C., and to Dr. I. F. Thompson, Snipes' Store, N. C. The first of these gentlemen is a retired physician, but desires to keep up with the science of medicine. The second is a promising young doctor with whom I was recently in consultation, and only had to mention it to get his name and money. So you see I have done twice as much as you asked for, and hope to be able to do more. * * * If my health improves

I will send you a contribution for THE RECORD. * * * If I never do, my heart is with you and THE RECORD.

With sentiments of high regard,

I am, as ever, your friend,

I. A. HANKS.

The above lines are taken from a letter, addressed to us from one of the many friends, developed by our position as an editor of a medical journal. We have received many such, and allude to them to express our thanks and gratification at these evidences of appreciation by the profession of the work in which we are engaged. In these, we find compensation for our labors and sacrifices, and encouragement in moments of gloom and despondency. They serve to soften and cultivate our hearts, and to draw our minds for the time from the toils and corroding cares of professional life, to the contemplation of that higher and better existence to which the true physician, in the conscious discharge of duty, may hopefully aspire.

Touching our age, we must ask the privilege of communicating that privately. As to our weight, we now tip the beam at two hnndred and thirty, and still growing, having gained one-half pound a month for the last twenty-eight years, and we feel confident, if such encouragement as our friend has given us shall continue to come, we shall continue to grow in a like ratio as long as we live, as that was the habit of our ancestors.

We shall, of course, take much pleasure in exchanging photographs with this noble and kind brother in the profession, and warmly reciprocate the expression of his desire to behold the shadow of a friend, whose real face he may never have the pleasure of seeing on earth; but whose prayer is that, under the blessing of Almighty God, he may have many years to come to reap a golden and glorious harvest; that he may see the wreath of fame that he has so long worn upon his brow (merited by his worthy son) and behold his name written among the few "who were never born to die."

"Like those who sink to rest,
With all their country's wishes blest."

And when the silver cord be loosed, or the golden bowl broken, may his spirit wing its way to that home "where many mansions be;" where is heard the song of Moses and the Lamb, and a "crown of righteousness" placed upon his brow, wreathed in Heaven's brightest jewels, and shineth with a lustre that fadeth not away. P.

